# Current knowledge on HIV-associated Plasmablastic Lymphoma

**DOI:** 10.4084/MJHID.2014.064

**Published:** 2014-11-01

**Authors:** Michele Bibas, Jorge J. Castillo

**Affiliations:** 1 Clinical Department, Hematology, National Institute for Infectious Diseases “Lazzaro Spallanzani” Rome, Italy.; 2 Division of Hematologic Malignancies, Dana-Farber Cancer Institute, Harvard medical School, Boston, USA.

## Abstract

HIV-associated PBL is an AIDS-defining cancer, classified by WHO as a distinct entity of aggressive DLBCL. To date less than 250 cases have been published, of them 17 are pediatric. The pathogenesis of this rare disease is related to immunodeficiency, chronic immune stimulation and EBV. Clinically is a rapid growing destructive disease mainly involving the oral cavity even if extraoral and extranodal sites are not infrequent. The diagnosis requires tissue mass or lymph node biopsy and core needle or fine needle biopsy is acceptable only for difficult access sites. Classically immunophenotype is CD45, CD20, CD79a negative and CD38, CD138, MUM1 positive, EBER and KI67 is >80%. Regarding the therapy, standard treatment is, usually, CHOP or CHOP-like regimens while more intensive regimens as CODOX-M/IVAC or DA-EPOCH are possible options. Use of cART is recommended during chemotherapy, keeping in mind the possible overlapping toxicities. Rituximab is not useful for this CD20 negative disease and CNS prophylaxis is mandatory. Intensification with ABMT in CR1 may be considered for fit patients. For refractory/relapsed patients, therapy is, usually, considered palliative, however, in chemo-sensitive disease, intensification + ABMT or new drugs as Bortezomib may be considered. Factors affecting outcome are achieving complete remission, PS, clinical stage, MYC, IPI score. Reported median PFS ranges between 6–7 months and median OS ranges between 11–13 months. Long term survivors are reported but mostly in pediatric patients. Finally, due to the scarcity of data on this subtype of NHL we suggest that the diagnosis and the management of HIV-positive PBL patients should be performed in specialized centers.

## History

This particular variant of diffuse large-B-cell lymphoma (DLBCL) was first recognized and described as a new entity in 1992 by Stein in the first edition of the textbook “Neoplastic Hematopathology”. 1 Delecluse and Stein in 1997, drawing from the consultation files of the lymphoma reference center at the Benjamin Franklin Hospital in Berlin, published the first case series of plasmablastic lymphoma (PBL) in HIV-positive (HIV+) patients in whom PBL was mainly located in the oral cavity.^2^

## Nomenclature

The term plasmablastic lymphoma (PBL) refers to a rare and distinct entity classified by the World Health Organization (WHO) as an aggressive subtype of DLBCL, characterized by a “diffuse proliferation of large neoplastic cells most of which resemble B-cell immunoblasts, but in which tumor cells have a plasma cell immunophenotype”.^3^ This disease is strongly associated with HIV infection, however it may also arise with other immunodeficiency states such as organ transplant patients, the elderly, and in immunocompetent individuals.^4^,^5^ HIV-associated PBL is included in the list of serious and life-threatening diseases that occur in HIV-positive individuals considered as “AIDS-defining” illnesses.^6^ When a person gets one of these illnesses, he or she is diagnosed with the advanced stage of HIV infection known as AIDS.

## Incidence and Temporal Trends

Since the introduction of combination antiretroviral therapy (cART), the incidence of AIDS-related lymphomas (ARLs), initially very high with 449 cases per 100.000 person-years (1996–2000 calendar period) and standardized incidence ratio (SIR) of 13.2, has dramatically declined. Nowadays, the incidence is near 194 cases per 100.000 person-years (2006–2010 calendar period) with SIR of 10.0.^7^ DLBCL remains the main type of cancer that develops in HIV-positive patients.^8^

The incidence of HIV-associated PBL accounts for approximately 2% of all ARLs. However because of the rarity, the exact incidence and temporal trends are unknown.^9^ Several case reports and case series have been published to date, accounting for no more than 250 cases. In a Pubmed search using the keywords “plasmablastic” and “lymphoma” and “HIV”, 313 articles were published between 2000 and 2009 (10 years), and 234 articles between 2010 and today (<5 years). It is unclear if the actual incidence of PBL has increased in recent years. This apparent increase in published case reports and series might just be a reflection of an increased awareness of PBL among clinicians.

## Pathogenesis

The pathogenesis of PBL HIV-associated is poorly understood and determined by the complexity of biological interplays between HIV-related immunodeficiency, genetic cellular abnormalities, co-infecting oncogenic viruses and chronic immune activation. In defining diagnostic and treatment strategies, it is of utmost importance to understand not only the molecular mechanism of viral carcinogenesis but also the host counterpart in term of immunologic status of the patient.^10^ The contribution of HIV to PBL pathogenesis might develop through four main mechanisms: 1) the duration and the degree of immunodeficiency or immunosuppression; 2) the induction of chronic antigenic stimulation leading to a chronic B-cell proliferation/exhaustion; 3) the loss of immune control of oncogenic herpesvirus as EBV; and 4) an incomplete immune reconstitution or factors unrelated to immune dysfunction.^11^

### Degree and duration of immunodeficiency

Time spent with higher viral load and lower CD4 counts plays an important role in the development of ARLs. HIV-positive PBL patients encompassing the pre- and post-ART eras have an average CD4 count at lymphoma presentation around 200 cell/mm3 and an average viral load of 250.000 copies/mL.^12^ The reported average duration from HIV diagnosis to PBL diagnosis is 8.9 years.^13^

### Chronic antigenic stimulation

HIV infection is characterized by chronic immune activation due to: 1) a persistent antigenic stimulation by viral HIV proteins or other viral co-infections (e.g. Cytomegalovirus and microbial product after microbial translocation in the gut); 2) the presence of a chronic general unresolved inflammatory state. This state can lead to a polyclonal B-cell expansion/dysfunction promoting the emergence of monoclonal B-cell, and to an abnormal production of stimulatory cytokines such IL-6.^14^

### Virology

HIV-associated ARL, including PBL, is strongly related to Epstein-Barr virus (EBV) infection, and near 80% of these lymphoma cells express EBNA1 and EBV-encoded RNAs detected using an in situ hybridization technique, representing then a type I latency pattern.^10^–^15^ Similarly, plasmacytoid Burkitt lymphomas express the same type I EBV latency pattern. In contrast in EBV-associated DLBCL immunoblastic lymphoma, tumour cells express EBV-encoded LMP1 (indicating a type II latency pattern). Of note, a subset of DLBCL may have a type III latency pattern as proved by the additional expression of the EBNA2 protein.^10^

### Molecular Genetics

The potential role of MYC gene rearrangements is currently unclear. This translocation mostly occurs in the context of complex karyotype abnormalities involving as a common partner in translocation the immunoglobulin gene. Of note in contrast of most lymphomas with MYC rearrangement that have a germinal center phenotype, PBL HIV+ have a typical postgerminal center profile, suggesting a distinct role in the pathogenesis.^10^ Of clinical interest, this translocation have been identified in near 50% of patients HIV+ with PBL and has been associated with worse outcome.^16^

## Clinical Presentations and Features

HIV-associated PBL is characterized for its predilection of involving the oral cavity as originally described. Nevertheless, near 45% of cases have been reported in extra-oral sites, including gastrointestinal tract, skin, soft tissue, heart, mediastinum, retroperitoneum, liver, lungs, testes, vulva, parotid gland, breast, central nervous system (CNS), lymph nodes and bone marrow.^13^–^17^ This lymphoma demonstrates a male predominance (4:1) with a median age at presentation of 40 years, being the disease the initial presentation of HIV infection in approximately 5% of the cases. Of note, also pediatric cases have been reported. Most patients present with rapid growing, sometimes destructive, disease in advanced clinical stage elevated LDH and B symptoms.^18^

## Diagnosis

Diagnosis requires a properly evaluated tissue biopsy of mass lesion or lymph node. Excisional biopsy is the gold standard; however, because frequently the site of the disease is difficult to access, core needle biopsy and fine needle aspiration (FNA) may be performed in conjunction with appropriate ancillary techniques for the differential diagnosis (i.e. flow cytometry, PCR for IgH and TCR gene rearrangements, FISH for major translocations, immunohistochemistry, cytogenetic studies, etc).

### Morphology

**Figure 1:**
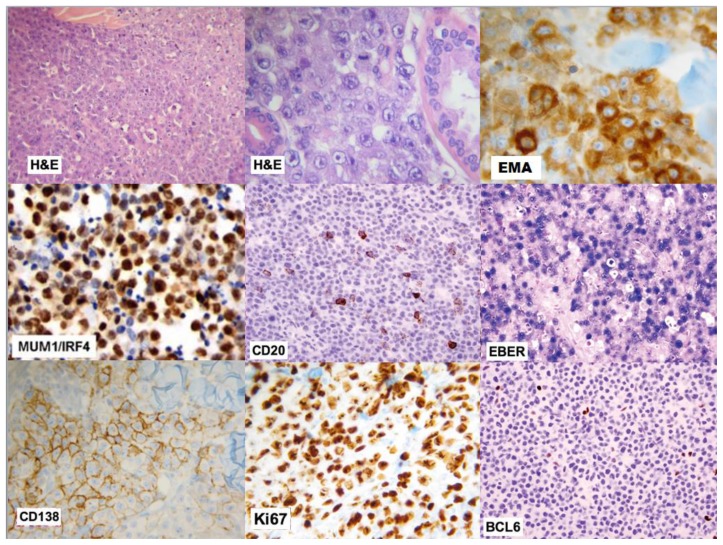
**H&E:** Large lymphoid cells with plasmablastic features, haematoxylin and eosin stain (medium and high resolution); **IRF4/MUM1:** multiple myelomas oncogene 1/interferon regulatory factor 4; **EMA**: epithelial membrane antigen; **KI-67%.** marker of the growth fraction; **EBER,** Epstein-Barr virus-encoded RNA; **CD20**, cluster of differentiation 20 (B-lymphocyte antigen);**BCL6,** B-cell lymphoma 6; **CD138**, cluster of differentiation 138 (syndecan-1)

PBL is characterized by monomorphic cellular proliferation of round to oval-shaped cells with either centrally or eccentrically placed nuclei and abundant eosinophilic cytoplasm in a diffuse sheet-like and cohesive growth pattern (Figure 1). Apoptotic bodies and mitotic figures are frequent, and tangible-body macrophages are easily detectable leading to a starry-sky appearance.^1^–^2^

### Immunophenotype

PBL is a high-grade B-cell lymphoma that arises from post-germinal center B-cell and usually express the characteristic immunophenotype of plasmacytoid terminally differentiated B-cell. As plasmablasts acquire plasma-cell markers (i.e. VS38c, CD38, MUM1/IRF4, CD138, EMA), they lose the leukocyte common antigen (CD45) and their B-cell markers CD20, CD79a, PAX5, and a high proliferation rate reflected by Ki67 expression >80%. (Figure 1). Cytoplasmic immunoglobulins are expressed in near 70% of cases. Interestingly, these lymphomas might express epithelial and endothelial markers such EMA and CD31, posing some problems in differential diagnosis with poorly differentiated solid tumors.^10^ Recently, an immunohistochemistry stain for PRDM1/BLIMP1 and XBP1 have been proposed to identify PBL, however this finding remains investigational.^11^

### Virology and genetics

HIV-associated PBL is closely linked to EBV infection, and more than 80% of PBL cells express EBV-encoded RNA (EBER-ISH). (Figure 1). MYC rearrangements, like other high-grade B-cell lymphomas of aggressive type, are found in near 50% and frequently correlated with EBV infection and worse prognosis. Molecular analysis of BCL2 and BCL6 are, usually, negative.^12^,^15^ The presence of HHV8 within the sample can indicate a different condition called HHV8-positive PBL arising from multicentric Castleman’s disease.^17^

### Clinical evaluation and staging

Patients with suspected HIV-associated PBL should have a complete medical history, a careful physical examination of node-bearing areas including Waldeyer’s ring, skin, liver and spleen, and an adequate evaluation of performance status and the presence of B symptoms. Laboratory evaluation should include a complete blood count, chemistry and immunoglobulins profile with *kappa* and *lambda* evaluation in plasma and urine. It is of utmost importance to evaluate renal, hepatic and cardiac functions prior intensive chemotherapy and patients should undergo echocardiographic evaluation of cardiac function because of the anthracycline–based chemotherapy, and gastrointestinal endoscopy if symptoms or lesions of suspicious on imaging studies are present. Pregnancy testing in women of childbearing age is mandatory if chemotherapy is planned.

Imaging studies should include whole body contrast-enhanced CT and an MRI of the head. FDG-PET in HIV associated lymphoma have a limitation in interpretation because the results can be confounded with inflammation and infections typically present in those patients.^26,.27^ A bone marrow biopsy and aspirate should be performed as involvement by PBL is found in up 25% of patients.^15^ Because PBL patients HIV+ are at high risk for presentation or recurrence in the CNS, particularly meninges, a lumbar puncture analyzing the cerebrospinal fluid by flow cytometry, cytology and molecular studies should be performed to check for leptomeningeal lymphoma. The Ann Arbor system is the most commonly used for staging purposes. We recommend the use of the International Prognostic Index (IPI) for risk-stratification.

### Differential diagnosis

The morphological differential diagnosis includes poorly differentiated and undifferentiated carcinoma, lymphoblastic lymphoma, plasmablastic variant of Burkitt’s lymphoma and anaplastic plasmacytoma. Differentiating PBLs from anaplastic plasmacytoma may be a diagnostic challenge. In fact highly aggressive plasma cell myeloma and extramedullary plasmacytoma may contain a predominance of plasmablasts with a similar immunophenotype to PBL, but myeloma cells are, usually, EBV negative. The presence high level of serum monoclonal proteins, and bone involvement with radiographically evident lytic lesions favor the diagnosis of myeloma rather than PBL. Plasma cell myeloma and plasmacytoma are very rare in the setting of HIV, and in the few cases reported in the literature a previous progression from MGUS was reported.^21^–^23^ However, the presence of MGUS is frequently encountered in patients at HIV presentation, reflecting dysregulation of the immune system due to the presence of HIV, but almost always MGUS disappears when viral load becomes undetectable^.24–25^ Other potential differential diagnoses include CD20-negative aggressive lymphomas such as solid or extracavitary primary effusion lymphoma and ALK+ DLBCL.

### HIV disease status

A detailed comprehensive evaluation of HIV disease must be performed including; duration of HIV infection, mode of HIV transmission, prior opportunistic infections, CD4+ cell count and viral load at HIV diagnosis and at diagnosis of lymphoma.^10^ A strategic history of HIV viral control and antiretroviral treatment with regards to HIV mutation and resistance should be assessed. In addition, information about HCV, HBV, tuberculosis, malaria, leishmaniasis coinfections and EBV, HHV8, CMV and sexually transmitted diseases are necessary for clinical management and therapeutic decisions.^14^

### Fertility preservation counselling and management

Fertility preservation is an essential consideration of cancer management. Because HIV-associated PBL frequently occur in young patients, this issue should be discussed as early as possible during treatment planning. For male patients, sperm banking should be planned before treatment initiation. Semen cryopreservation is recommended independently of patient’s age, according to their wishes of future paternity. Embryo or oocyte cryopreservation can address the sterility chemo-radiotherapy induced in women, this method it actually the gold standard to preserve female fertility, but requires in vitro fertilization procedure. Freezing ovarian tissue before treatment may be an option (the tissue is harvested with laparoscopy and re-implanted after thawing in the pelvis when needed) but is an experimental method.^28^

## Management

### First –line treatment

The treatment of PBL HIV-associated has not been standardized as prospective studies to define a standard of care are lacking. It is a common practice to start patients on combination chemotherapy. In the recently updated National Comprehensive Cancer Network guidelines, the recommendation is to treat HIV+ PBL with intensive regimens as CODOX-M/IVAC, Hyper-CVAD, or DA-EPOCH as possible options. Standard CHOP seems not an adequate therapy.^29^ However, CHOP therapy is often given to treat PBL.^14^,^20^ Intensification of induction chemotherapy with autologous bone marrow transplantation (ABMT), thought to be a good option in HIV-negative patients with chemosensitive disease, has also been shown to be feasible also in HIV+ patients.^9^,^30^ Of note, HIV infection alone should not preclude an attempt to obtain stem cell in candidates for ABMT. Chemotherapy plus G-SCF seems to mobilize better than G-CSF alone, and at least 3g/m^2^ of cyclophosphamide is recommended.^31^

### New drugs

Because PBL shares many morphologic and immunophenotypic traits with plasmablastic myelomas some studies have reported that the proteasome inhibitor bortezomib alone or in combination with chemotherapy may have an antitumor effect in PBL, blocking NF*_K_*B or overcoming the typical chemoresistance of this disease. For the same reason, the use of lenalidomide has been reported in PBL. This well-known immunomodulatory agent has proved to be effective as a single agent in aggressive relapsed or refractory HIV-negative patients with non-Hodgkin’s lymphoma by enhancing the immune system, with robust response rates. However, the reported outcome, at the case report level, with these new agents are transient.^32^,^33^ Due to the lack of CD20 expression, the use of the anti-CD20 monoclonal antibody rituximab, is unlikely to be of benefit, however it could be considered if partial expression of CD20 is detected within the malignant cells.

### Refractory or relapsed patients

Treatment in patients with refractory or relapsed HIV-associated PBL is considered palliative although some cases of long-term survival have been described. In general, a more intensive chemotherapy is planned for relapsed patients, and for fit patients intensification of chemotherapy with AMBT may be an option. Multiple reports from single centers or cooperative groups have been published. Effectiveness of such therapy was not significantly different between HIV-positive and HIV-negative patients, in term of treatment-related mortality, opportunistic infections, immune recovery, and OS. Of note, allogeneic BMT is a more limited option in HIV-positive relapsed PBLs.^9^,^30^

## Specific Treatment Considerations

### Supportive therapy

More vigorous supportive care is necessary for HIV-infected patients than in patients who are not infected with HIV, and antibacterial, antifungal, and antiviral prophylaxis may be offered in accordance with current guidelines.^30^,^32^ Patients should be screened for hepatitis B infection and antiviral prophylaxis initiated if indicated. CD4 count cell must be regularly evaluated during and after chemotherapy, and cotrimoxazole prophylaxis strongly recommended when the CD4 cell count falls below 200 cell/ml for prevention of *Pneumocystis* pneumonia.

### CNS prophylaxis

Patients with PBLs HIV are at risk of for leptomeningeal disease. Considering this high risk of progression during the treatment or recurrence during the remission, the use of intrathecal prophylaxis is considered a mandatory part of the systemic treatment.^34^ Controlled studies on this field are not available, so the standard procedure has not been defined. However, intrathecal methotrexate or cytarabine are administered at each cycle of chemotherapy, based upon institutional preference.^35^

### Use of cART during chemotherapy

Most guidelines recommend, on the basis of different meta-analyses, the use of cART during chemotherapy. However, it is important to keep in mind the possible overlapping toxicity, pharmacokinetic interactions, and adherence problems, to avoid stop and start cART strategy because of HIV drug resistance.^10^,^14^ Although no prospective studies have been performed, and controversies abound, the addition of cART to chemotherapy seems to have a favorable effect and gives a benefit both on response and survival.^30^,^37^ A potential explanation for this finding may be that the use of antiretroviral therapy can restore immune surveillance allowing for more efficient anti-cancer effect.^38^

## Prognostic Factors

Since the introduction of cART, the prognosis of patients with HIV-associated aggressive lymphoma who receive optimal therapy has markedly improved, and now the outcome is near the same of the HIV-negative counterpart. In general, CD4+ cell counts >200 cells/mm^3^ and low IPI scores are independent positive prognostic factors.^10^,^14^ In patients on effective cART (i.e. undetectable viral load, high CD4, low incidence of comorbidities), HIV-related scores are less important prognostic factors than lymphoma related features (i.e. histology, tumor burden, LDH, performance status).^20^ The prognosis of HIV-associated PBL remains poor. In the recent literature, the median progression-free (PFS) and overall survival (OS) ranged between 6–7 months and 11–13 months respectively, without statistical difference between patients treated with CHOP or CHOP-like regimens and more intensive therapy.^15^ However, recently a trend for higher response rates and longer OS have been reported with cases of long-term survivors reported in the literature.^9^,^39^ Several factors affecting outcome are reported in the literature; however, the most important are achieving complete remission, performance status, clinical stage, MYC gene rearrangements, and aaIPI.^39^ Furthermore, extended and destructive masses at diagnosis and comorbidities might confer an adverse prognosis.

### Differences between HIV-positive and negative PBLs

While data on HIV-negative PBLs patients are sparse, several differences have been identified.^4^,^5^ HIV-negative PBL occurs in older patients and affects relatively more females. HIV-negative PBL is much more heterogeneous in terms of stage at time of diagnosis with extra-oral involvement being reported at a higher frequency.^9^ Immunosuppression is the major risk factor for development of HIV-negative PBL, with post-transplant lymphoproliferative disease comprising nearly half of the reported cases. In a literature review, HIV-negative PBL showed to have worse outcome than patients with HIV-positive PBL with a median OS of nine months; CR after induction chemotherapy being the only prognostic factor associated with improved outcomes.^40^

### Pediatric cases

Although the majority of patients are adults, PBL has also been reported in pediatric HIV-infected patients.^4^,^5^ A literature review identified only 17 cases. The median age was ten years (range 2–17), >80% with advanced stage at presentation and jaw/oral cavity as the most common site of initial disease.^41^ However, extranodal locations (e.g. skin, vulva, spine, scalp) have also been reported. Prognosis is, usually, poor with two reported long-term survivors (3.5 and eight years). ^41,42.^

## Conclusions

IV-associated PBL is an aggressive and rare subtype of NHL with an aggressive clinical course and poor outcomes. The current knowledge on this rare lymphoma is described in this review and summarized for rapid consultation in Table 1. Finally, the evidence supporting all the strategies reported here arises from single-center series and reviews and not from prospective randomized trials. Hence, due to the scarcity of data on this subtype of NHL and until more definitive evidence become available, the diagnosis and management of HIV-positive PBL patients should be performed in specialized centers.

## Figures and Tables

**Table 1:** Key Points on HIV-associated Plasmablastic lymphoma

HIV-associated PBL is an AIDS-defining cancer, classified by WHO as a distinct entity of aggressive DLBCL. Less than 250 cases published to date, 17 in the pediatric population. Pathogenesis related to immunodeficiency, chronic immune stimulation, oncogenic herpesvirus as EBV. Clinical: rapid growing destructive disease mainly of the oral cavity, frequently also extraoral extranodal sites. Diagnosis requires tissue mass or lymph node biopsy. Core needle od fine needle biopsy for difficult access sites Immunophenotype usually CD45, CD20, CD79a negative and CD38, CD138, MUM1 positive, EBER and KI67 >80% Frequently diagnosed with low CD4+ and high viral load. Recently also reported in effective cART and high CD4. Treat with regimen more intensive than CHOP, consider intensification with ABMT in CR1 for fit patients. Rituximab not useful (CD20-), Bortezomib used at case report level, with transient response. CNS prophylaxis mandatory. Use of cART recommended during chemotherapy. Keep in mind possible overlapping toxicity. For refractory/relapsed patients consider palliative therapy, chemosensitive disease intensification+ABMT or new drugs Factors affecting outcome: achieving complete remission, PS, Clinical stage, MYC, IPI. Median PFS ranges 6–7 months; Median OS ranges 11–13 months. Long term survivor reported, mostly in pediatric patients.
